# Intra- and interobserver variability of language mapping by navigated transcranial magnetic brain stimulation

**DOI:** 10.1186/1471-2202-14-150

**Published:** 2013-12-05

**Authors:** Nico Sollmann, Theresa Hauck, Alexander Hapfelmeier, Bernhard Meyer, Florian Ringel, Sandro M Krieg

**Affiliations:** 1 Department of Neurosurgery, Klinikum rechts der Isar, Technische Universität München, Ismaninger Str. 22, 81675 Munich, Germany; 2 Institute of Medical Statistics and Epidemiology, Technische Universität München, Ismaninger Str. 22, 81675 Munich, Germany

**Keywords:** Cortical mapping, Language, Transcranial magnetic stimulation, Navigated brain stimulation, Object naming

## Abstract

**Background:**

Repetitive navigated transcranial magnetic stimulation (rTMS) has been used for studying language organization in healthy volunteers and patients, and to detect cortical areas involved in language processing. However, little is known about the reliability of this method. To determine the reliability of rTMS language mapping, we conducted both an interobserver and an intraobserver investigation.

**Methods:**

Ten right-handed healthy subjects underwent language mapping by rTMS and the same object-naming task three times. Intraobserver and interobserver reliability of seven different error types were tested by two investigators. Analysis was performed blinded to the previous results and stimulated cortical sites.

**Results:**

Overall, the results of both the interobserver and the intraobserver investigations show variable accordance. This is demonstrated by comparing the error rates of all different error types of the three examinations. Considering the most important error type, “no response”, there is only small variability in inter- and intraobserver mapping.

**Conclusions:**

With our current protocol, interobserver and intraobserver comparisons only corresponded partially. Thus, although rTMS seems a promising method for preoperative planning as well as neuropsychological research, the current protocol needs further improvement.

## Background

Data on the cortical organization of human language is largely based on functional MRI (fMRI) and magnetoencephalography (MEG) studies [1-6]. Moreover, intraoperative language mapping by bipolar direct cortical stimulation (DCS) during awake surgery for the left [7-9] and right hemispheres [10] also contributed to the current knowledge. Although DCS mapping is highly reliable, it does not allow for analysis of cortical language distribution in the healthy brain. Therefore, as the second lesion-based but non-invasive modality, transcranial magnetic stimulation (TMS) was used since two decades to disrupt language function [11-13]. With the introduction of navigated TMS (nTMS) in neuroscience in the last years repetitive nTMS (rTMS) was also increasingly used for mapping of cortical regions, which are presumably language-eloquent [11,12,14-16].

In combination with an object naming task, rTMS was also compared to intraoperative DCS during awake surgery [15,16]. Therefore, this new technique is already seen as a tool for preoperative mapping of cortical language function. However, while mapping of the primary motor cortex by nTMS via provoking motor evoked potentials was already proven to be sufficiently reproducible [17,18], data confirming the reliability of rTMS for language mapping are still lacking.

Given the remaining uncertainty about reproducibility of rTMS language mapping, this study aims to determine the reliability of rTMS language mapping by conducting both an intra- and interobserver investigation. To mirror the current practice of rTMS language mapping we strictly adhered to the recently published protocols instead of using a new and more standardized protocol [15,16,19].

## Methods

### Study design

The study was designed as prospective, non-randomized.

### Study subjects

Ten purely right-handed (according to the Edinburgh handedness questionnaire), monolingual and healthy volunteers underwent rTMS three times. German was the only primary language for all volunteers. Five subjects were female and five were male. The median age was 24.2 years (range 22.7 to 30.3 years). No one was under any kind of medication.

The inclusion criteria were German as mother tongue, right handedness, written informed consent, and age above18 years. The exclusion criteria were general rTMS exclusion criteria (pacemaker, cochlear implant), previous seizures, second mother tongue, bilateral handedness, and pathological findings on cranial MRI.

### Ethics

The experimental protocol was approved by the ethical committee of the Technical University of Munich (registration number: 2793/10) in accordance with the Declaration of Helsinki. All volunteers provided written informed consent prior to MR imaging.

### Navigational MRI

Prior to the first rTMS mapping session, all volunteers underwent a navigational MRI scan on the same clinical 3 Tesla MR scanner (Achieva 3T, Philips Medical Systems, The Netherlands B.V.) by use of an 8-channel phased array head coil. A 3D gradient echo sequence (TR/TE 9/4 ms, 1 mm^2^ isovoxel covering the whole head, 6 minutes 58 seconds acquisition time) without intravenous contrast administration was used for anatomical co-registration. Then, by using the DICOM standard, the three-dimensional dataset was transferred to the rTMS system.

### rTMS language mapping

#### Experimental setup

Each subject underwent rTMS language mapping three times by two different investigators. In order to evaluate the intraobserver reliability, the first and the second mapping were conducted by the same investigator. Another investigator performed the third mapping to determine interobserver reliability. The time lag between the first and the second mapping was 191.4 ± 67.5 days. Between the first and the third mapping, the time lag was 211.8 ± 60.7 days. Data collection and analysis were performed blinded to previous results, by the same investigator who also performed the mapping.

The experimental setup was strictly the same for all cortical language mappings, which were performed with the Nexstim eXimia NBS system version 4.3 and the NexSpeech® module (Nexstim Oy, Helsinki, Finland), as reported earlier [15,16]. In short, object presentation was performed by 131 color pictures of common objects, displayed at an inter-stimulus interval (ISI) of 2.5 s on a video screen in front of the volunteer. The subject was instructed to name the objects in German as quickly and precisely as possible. Magnetic pulses were applied 300 ms after the picture presentation onset, according to our current knowledge of the timing of naming-related activity reported in MEG and TMS studies [5,20] and according to a protocol established in the first study on navigated TMS for language mapping [14]. First, baseline testing of all objects without stimulation was performed twice prior to each language mapping. All objects, which were misnamed at least once, were discarded from the stimulus sequence and the number of baseline errors was documented. Subsequently, the definite diagnostic naming task was presented time-locked to a train of rTMS pulses. During baseline, as well as during language mapping, minimum electric field strength of 55 V/m at the cortical surface was accepted. Overall, field strength ranged between 55–80 V/m among all subjects. Frequency and intensity of the rTMS were personalized based on a previously published protocol at 100% Resting Motor Threshold (RMT) of the left-sided cortical hand area of the right abductor pollicis brevis muscle [21,22]. Trains of 5–7 TMS bursts were administered to vPrG and opIFG with three different setups (a: 5 Hz, 5 pulses; b: 7 Hz, 5 pulses; c: 7 Hz, 7 pulses). Then, the setup (a–c) that caused the most effective language impairment was used for further mapping. If there was no clear difference in the effect on language the most comfortable frequency was chosen. If no evident responses were obtained, the intensity was increased to 110–120% RMT and we started again with setup a–c. For objective and detailed analysis, the object naming baseline performance, as well as the mapping session, were digitally video-recorded [14,15].

#### Language mapping procedure

The stimulation coil was randomly moved during the ISI in 10 mm steps over the left hemisphere. In order to achieve maximum field induction, the coil was placed tangential to the skull and with the field in an anterior-posterior orientation, as reported earlier [12,14,23]. All sites were stimulated three times each, and the same sites were not targeted consecutively. Language mapping of the left hemisphere required up to 60 minutes per subject. Directly after each mapping, each volunteer was asked to determine the rate of discomfort or pain according to the visual analogue scale (VAS) from 0 (no pain) to 10 (maximum pain) both for temporal regions and for the convexity (defined as the lateral, dorsal, rostral, and cranial surface of the brain without the region covered by the temporal muscle).

### Data analysis

The mapping data of all subjects were examined as previously described [14,15]. The first two mappings of each volunteer were analyzed by the same examiner who had also performed the corresponding stimulation sessions. The third mapping was evaluated by the second examiner who conducted the third mapping sessions in an analogue way. The comparison between the first and second mapping sessions was intended to gauge intraobserver variability, while the comparison between the second and third mapping sessions measures interobserver variability. Analysis was performed strictly blinded to the previous results and stimulated cortical sites.

Prior to video analysis, the individual RMT and rTMS frequencies were documented. Then, the video was analyzed for any disturbance of language processing compared to the baseline response. During video analysis, the cortical stimulation sites were hidden. Errors related to direct stimulation of muscles or associated with pain were discarded from the analysis. All other observed errors were categorized following a rule presented in Corina et al. [24]:

No-response errors: stimulation leads to a complete lack of naming response.

Performance errors: form-based distortions that are slurred, stuttered, or imprecisely articulated. This category contains both dysarthric and apraxic language production errors.

Hesitation: delayed onset of naming compared to baseline.

Neologisms: form-based errors, which are possible but nonexistent words. For example, the target word “horse” is replaced with the word “herp”.

Semantic paraphasias: errors in which the volunteer substitutes a semantically related or associated word for the target word. For example, the target word “cow” is replaced by the word “horse”.

Phonologic paraphasias: characterized by unintended phonemic modification of the target word. The spoken word resembles the target word, but is phonetically different. For example, the target word “pants” is replaced with “plants”.

Circumlocution errors: errors in which the subject talks about or “around” the target, instead of naming it. For example, the target word “chair” is replaced with “sit down”, explaining the use of the target word.

### Frequency map of evoked errors

#### Anatomical localization

**Figure 1:**
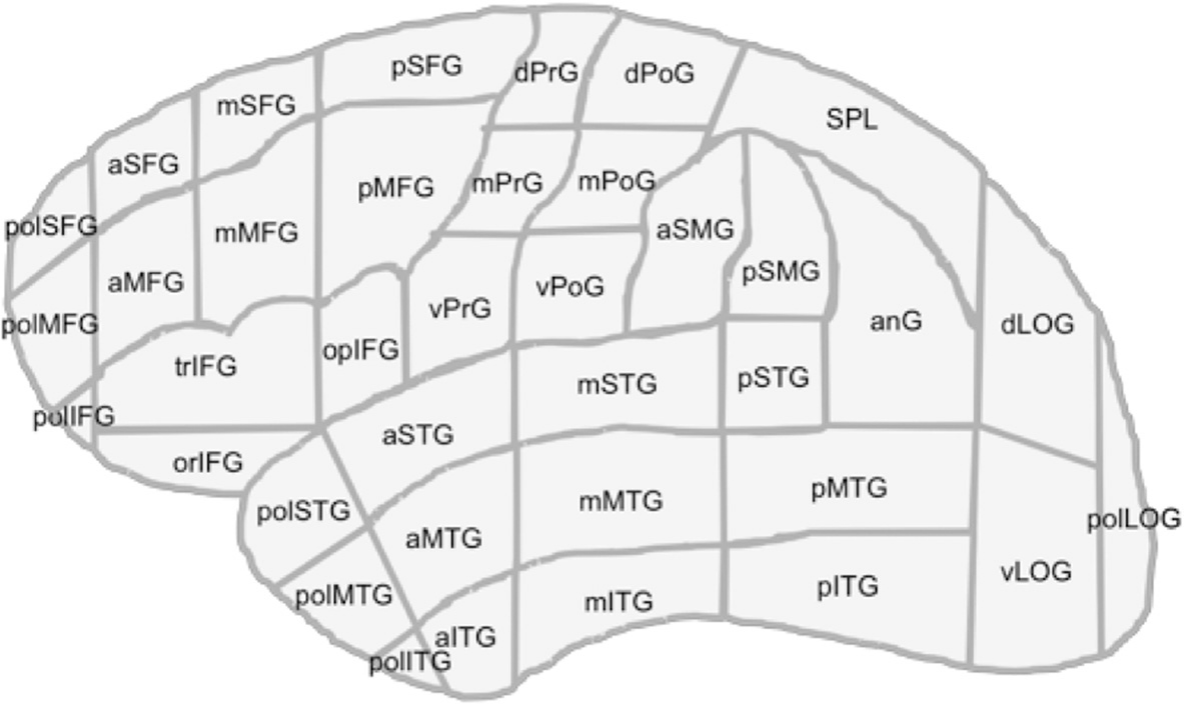
**Cortical parcellation system.** Anatomical areas, as described in Corina et al. [25].

The location of stimulation in the rTMS system was determined with a real time physics–modeling system (eXimia 4.3, Nexstim Oy, Helsinki, Finland). This system calculates the intracranial location of stimulation induced by the coil and displays this information on a high-resolution 3D MRI. As a result, the effects of the stimulation train can be pinpointed to an anatomical location when the coil position is tracked. According to an approach for evaluating effects of intraoperative language mapping on an anatomical level, we used the cortical parcellation system (CPS) for further anatomy-related data analysis and visualization [24,25]. The CPS divides the cortex into 37 anatomical regions for evaluation of the anatomical site of the stimulation (Table 1). The cortical gyri belonging to these anatomical CPS subregions were identified from 3D MRIs and the regions were drawn on a 3D image [25]. Then, the rTMS data were projected on the parcellated cortex. This approach allows a statistical analysis of error frequency and comparison of the data, both between individual volunteers and over the entire studied population. The locations of these regions were displayed on an anatomical brain template (Table 1, Figure 1).

**Table 1:** Cortical parcellation system

**Abbreviation**	**Anatomy**
aITG	Anterior inferior temporal gyrus
aMFG	Anterior middle frontal gyrus
aMTG	Anterior middle temporal gyrus
anG	Angular gyrus
aSFG	Anterior superior frontal gyrus
aSMG	Anterior supramarginal gyrus
aSTG	Anterior superior temporal gyrus
dLOG	Dorsal lateral occipital gyrus
dPoG	Dorsal post-central gyrus
dPrG	Dorsal pre-central gyrus
mITG	Middle inferior temporal gyrus
mMFG	Middle middle frontal gyrus
mMTG	Middle middle temporal gyrus
mPoG	Middle post-central gyrus
mPrG	Middle pre-central gyrus
mSFG	Middle superior frontal gyrus
mSTG	Middle superior temporal gyrus
opIFG	Opercular inferior frontal gyrus
orIFG	Orbital part of the inferior frontal gyrus
pITG	Posterior inferior temporal gyrus
pMFG	Posterior middle frontal gyrus
pMTG	Posterior middle temporal gyrus
polIFG	Polar inferior frontal gyrus
polITG	Polar inferior temporal gyrus
polLOG	Polar lateral occipital gyrus
polMFG	Polar middle frontal gyrus
polMTG	Polar middle temporal gyrus
polSFG	Polar superior frontal gyrus
polSTG	Polar superior temporal gyrus
pSFG	Posterior superior frontal gyrus
pSMG	Posterior supramarginal gyrus
pSTG	Posterior superior temporal gyrus
SPL	Superior parietal lobe
trIFG	Triangular inferior frontal gyrus
vLOG	Ventral lateral occipital gyrus
vPoG	Ventral post-central gyrus
vPrG	Ventral pre-central gyrus

Anatomical names and abbreviations according to Corina et al. [25].

Additionally, for combined statistical and anatomical analysis, two parcellation groups were formed:

(1) Anterior group: including CPS regions opIFG, trIFG, and vPrG

(2) Posterior group: including CPS regions AnG, aSMG, pSMG, mSTG, and pSTG

#### Stimulation assessment

As aforementioned, each stimulation site was stimulated three times during rTMS language mapping. To determine whether an individual brain region gave rise to language deficits during rTMS or not, the following definitions for region positivity and negativity were used:

(1) Positive brain region: a region was considered to give rise to language deficits if any of the trains delivered to the region elicited naming errors, regardless of the error type; and,

(2) Negative brain region: a brain region was considered not to give rise to language deficits if the region had been stimulated with at least one stimulation train and no language deficits of any error type were generated.

### Statistics

Descriptive and explorative statistics were used for data analysis. The distribution of quantitative data is presented by the mean ± standard deviation. Differences concerning the determined RMT, the mapping intensity, and the indicated pain during the three mappings were tested via analysis of variance (ANOVA) for repeated measures (SigmaStat 3.5, Jandel Scientific, Erkrath, Germany). Additionally, a permutation test of symmetry was performed to evaluate differences in the utilized frequencies and pulses (The R Foundation for Statistical Computing, Vienna, Austria). A *P* value of *P* < 0.05 was considered significant.

The concordance correlation coefficient (CCC) was used to compute intra- and inter-observer reliability (The R Foundation for Statistical Computing, Vienna, Austria) [26]. For the former, two assessments of one observer were compared. For the latter, the first measurements of observer one were compared against the ones of observer two.

The CCC measures the agreement between two variables. Perfect agreement means a CCC of 1.

## Results

### Healthy subjects

Altogether, 10 volunteers were enrolled (Table 2). No volunteer had to be excluded due to intracranial pathologies.

**Table 2:** Stimulation parameters

		**1st mapping**	**2nd mapping**	**3rd mapping**	**p**
Pain (VAS)	convexity	1.7 ± 1.2	2.1 ± 1.2	2.0 ± 1.2	0.662
(Mean ± SD)	temporal	5.5 ± 1.6	5.7 ± 1.8	4.8 ± 1.5	0.318
motor threshold (% Output) (mean ± SD)		37.8 ± 7.0	36.7 ± 5.4	35.9 ± 7.1	0.439
mapping intensity (% MT) (mean ± SD)		102 ± 6	104 ± 8	102 ± 6	0.685
most comfortable	5 Hz, 5 Pulses	5	3	2	0.233
	7 Hz, 5 Pulses	2	6	6	
	7 Hz, 7 Pulses	3	1	2	

Stimulation parameters used in the study including pain score according to the visual analogue scale (VAS). RMT = resting motor threshold (stimulator output); Hz = stimulation train frequency; # pulses = number of pulses in train; int % = stimulation intensity (of maximum stimulator output).

### Stimulation-related discomfort

Each stimulation session was generally well tolerated by the volunteers and no volunteers requested reduction of the stimulation intensity due to pain. The mean VAS score for maximum painful stimuli was comparable in both groups (Table 2). However, given experiences in former studies, the spatial extent of stimulation had to be restricted because of unacceptable pain in orIFG, polSTG, polMTG, and aMTG. In addition, the ITG was not mapped due to the distance between skin and brain, and the consequent too-low stimulation intensity.

### Distribution of the different error types

#### First mapping

##### Sum of all errors

**Figure 2:**
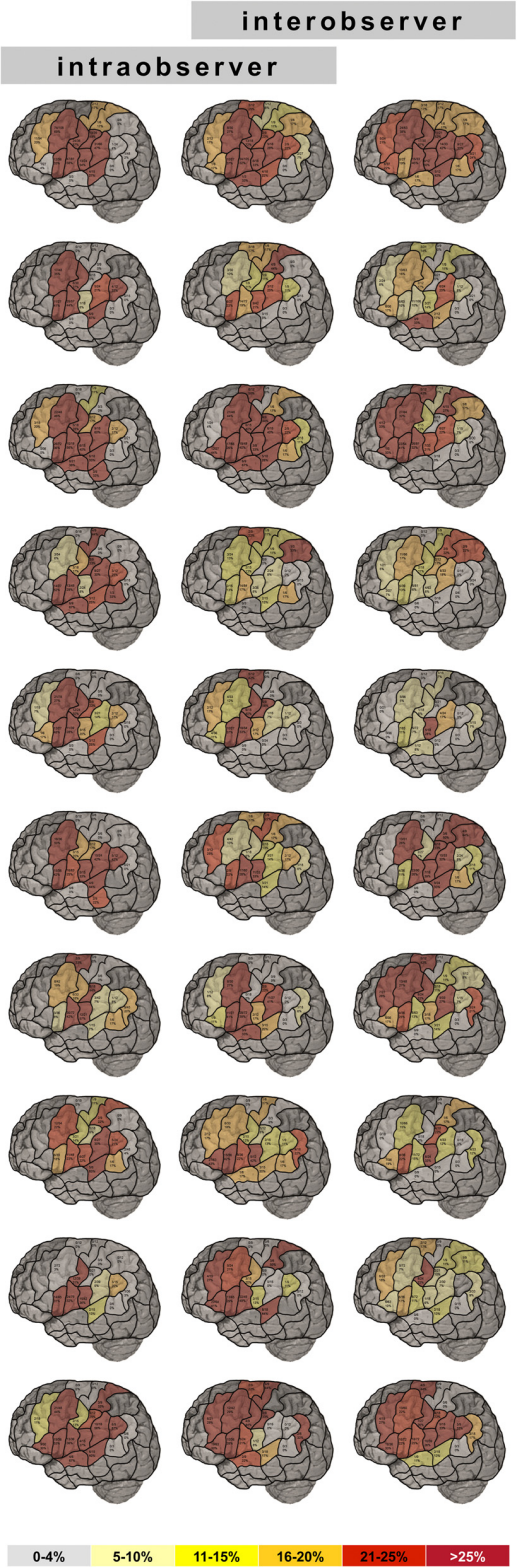
**All errors.** Language mapping by rTMS presented with the cortical parcellation system. Each line shows the results for all errors of one subject. The left and the intermediate column illustrate intraoberserver, the intermediate and the right column illustrate interobserver variability.

Overall, 3606 stimulations were performed. Of these, 961 stimulations elicited a language error. This equals an error rate of 26.7% (Table 3). The errors were mainly located within the classical Broca’s area of the opIFG and its surrounding structures. However, when also considering dysarthria, vPrG and mPrG showed relatively high error rates (Figure 2).

**Table 3:** First mapping

**Subject**	**No response**		**Performance**		**Hesitation**		**Neologism**		**Phonological**		**Circumlocution**		**Semantic**		**Totals**		
	**Errors**	**Rate**	**Errors**	**Rate**	**Errors**	**Rate**	**Errors**	**Rate**	**Errors**	**Rate**	**Errors**	**Rate**	**Errors**	**Rate**	**Errors**	**Trials**	**Error rate**
M1	28	0.05	124	0.21	40	0.07	48	0.08	0	0.00	0	0.00	1	0.00	241	591	0.41
M2	17	0.06	28	0.10	32	0.11	0	0.00	0	0.00	0	0.00	0	0.00	77	294	0.26
M3	22	0.06	42	0.12	32	0.09	5	0.01	0	0.00	0	0.00	2	0.01	103	363	0.28
M4	5	0.02	32	0.11	20	0.07	3	0.01	1	0.00	0	0.00	2	0.01	63	300	0.21
M5	2	0.01	51	0.17	14	0.05	3	0.01	2	0.01	0	0.00	0	0.00	72	300	0.24
F1	17	0.06	37	0.13	22	0.08	2	0.01	0	0.00	0	0.00	2	0.01	80	291	0.27
F2	20	0.05	31	0.08	34	0.09	0	0.00	0	0.00	0	0.00	0	0.00	85	366	0.23
F3	10	0.03	19	0.05	32	0.09	1	0.00	0	0.00	0	0.00	1	0.00	63	354	0.18
F4	16	0.04	35	0.08	21	0.05	0	0.00	1	0.00	0	0.00	2	0.00	75	450	0.17
F5	42	0.14	11	0.04	47	0.16	0	0.00	0	0.00	0	0.00	2	0.01	102	297	0.34
MIN	2	0.01	11	0.04	14	0.05	0	0.00	0	0.00	0	0.00	0	0.00	63	291	0.17
MAX	42	0.14	124	0.21	47	0.16	48	0.08	2	0.01	0	0.00	2	0.01	241	591	0.41
MEDIAN	17	0.05	33.5	0.11	32	0.09	1.5	0.01	0	0.00	0	0.00	1.5	0.00	78.5	327	0.25

Distribution of naming errors in the first mapping: Summary of naming errors induced by rTMS per subject (M1-M5: male volunteers; F1-F5: female volunteers). Below is reported number of trials (rTMS trains), total number errors, and error type and rate of each given error type of all induced errors.

##### No response errors

Of 3606 left hemispheric stimulations, 179 no response errors occurred. This is equal to an error rate of 5.0% (Table 3). No response errors were observed in all subjects during the first mapping session.

##### Performance errors

Of the 3606 stimulations, 410 performance errors were observed, which is equal to an error rate of 11.4% (Table 3). Each volunteer showed performance errors.

##### Hesitation errors

Hesitation errors as an isolated error type were very widely distributed and were observed in all volunteers; 294 hesitation errors were found out of 3606 stimulations, which is equal to an error rate of 8.2% (Table 3).

##### Other errors

Out of all 3606 stimulations, 62 neologisms (error rate 1.7%), and 4 phonological errors (error rate 0.1%) occurred (Table 3). There were also 12 semantic errors (error rate 0.3%). No circumlocutions were observed.

#### Second mapping

##### Sum of all errors

During the second mapping sessions, the left hemisphere was stimulated between 279 and 348 sites (median: 297.0 sites). Across all subjects, 3033 total stimulations were performed in the left hemisphere. Of these, 705 stimulations elicited a language error, for an error rate of 23.2% (Table 4). These errors were again mainly located within the classical Broca’s area of the opIFG, as well its surrounding structures. Again, with regard to elucidating dysarthria, vPrG and mPrG showed relatively high error rates (Figure 2).

**Table 4:** Second mapping

**Subject**	**No response**		**Performance**		**Hesitation**		**Neologism**		**Phonological**		**Circumlocution**		**Semantic**		**Totals**		
	**Errors**	**Rate**	**Errors**	**Rate**	**Errors**	**Rate**	**Errors**	**Rate**	**Errors**	**Rate**	**Errors**	**Rate**	**Errors**	**Rate**	**Errors**	**Trials**	**Error rate**
M1	17	0.05	34	0.10	41	0.12	4	0.01	0	0.00	0	0.00	0	0.00	96	345	0.28
M2	11	0.04	7	0.03	28	0.10	0	0.00	0	0.00	0	0.00	0	0.00	46	279	0.16
M3	25	0.07	29	0.08	50	0.14	4	0.01	0	0.00	0	0.00	4	0.01	112	348	0.32
M4	2	0.01	18	0.06	12	0.03	1	0.00	0	0.00	0	0.00	1	0.00	34	288	0.12
M5	7	0.02	32	0.11	15	0.05	3	0.01	0	0.00	0	0.00	1	0.00	58	288	0.20
F1	4	0.01	26	0.09	22	0.08	0	0.00	0	0.00	0	0.00	1	0.00	53	282	0.19
F2	10	0.03	18	0.06	43	0.14	0	0.00	0	0.00	0	0.00	0	0.00	71	303	0.23
F3	11	0.04	21	0.07	40	0.13	3	0.01	0	0.00	0	0.00	4	0.01	79	306	0.26
F4	13	0.04	42	0.14	27	0.09	0	0.00	0	0.00	0	0.00	1	0.00	83	297	0.28
F5	16	0.05	14	0.05	42	0.14	0	0.00	0	0.00	0	0.00	1	0.00	73	297	0.25
MIN	2	0.01	7	0.03	12	0.03	0	0.00	0	0.00	0	0.00	0	0.00	34	279	0.12
MAX	25	0.07	42	0.14	50	0.14	4	0.01	0	0.00	0	0.00	4	0.01	112	348	0.32
MEDIAN	11	0.04	23.5	0.08	34	0.11	0.5	0.00	0	0.00	0	0.00	1	0.00	72	297	0.24

Distribution of naming errors in the second mapping (first remapping): Summary of naming errors induced by rTMS per subject (M1-M5: male volunteers; F1-F5: female volunteers). Below is reported number of trials (rTMS trains), total number errors, and error type and rate of each given error type of all induced errors.

##### No response errors

Altogether, 116 no response errors occurred. For the number of given stimulations, we obtained an error rate of 3.8% (Table 4). In the second mapping, no response errors were observed in all subjects as well.

##### Performance errors

Within all volunteers, 241 performance errors were elicited during 3033 stimulations, resulting in an error rate of 7.9% (Table 4). Again, each volunteer presented performance errors.

##### Hesitation errors

320 hesitation errors were elicited, for an error rate of 10.6% (Table 4). Hesitation errors were observed in all subjects.

##### Other errors

With regard to all volunteers, 3033 left hemispheric stimulations elicited 15 neologisms (error rate 0.5%) and 13 semantic errors (error rate 0.4%). Neither phonological errors nor circumlocutions were observed (Table 4).

#### Third mapping

##### Sum of all errors

The left hemisphere was stimulated between 384 and 477 sites (median: 436.5 sites) during the third mapping sessions. With 4344 total stimulations, the highest number of stimulations was set during these third mapping sessions. Thereby, 745 language errors were elicited (error rate 17.2%; Table 5). Once again, these errors of any kind were primarily located within the classical Broca’s area of the opIFG, as well its surrounding structures (Figure 2).

**Table 5:** Third mapping

**Subject**	**No response**		**Performance**		**Hesitation**		**Neologism**		**Phonological**		**Circumlocution**		**Semantic**		**Totals**		
	**Errors**	**Rate**	**Errors**	**Rate**	**Errors**	**Rate**	**Errors**	**Rate**	**Errors**	**Rate**	**Errors**	**Rate**	**Errors**	**Rate**	**Errors**	**Trials**	**Error rate**
M1	31	0.08	40	0.10	46	0.12	11	0.03	2	0.01	0	0.00	1	0.00	131	384	0.34
M2	9	0.02	2	0.00	44	0.09	0	0.00	0	0.00	0	0.00	1	0.00	56	468	0.12
M3	30	0.07	6	0.01	66	0.15	3	0.01	1	0.00	0	0.00	1	0.00	107	441	0.24
M4	3	0.01	2	0.00	35	0.07	1	0.00	0	0.00	0	0.00	2	0.00	43	468	0.09
M5	2	0.00	2	0.00	21	0.05	5	0.01	0	0.00	0	0.00	0	0.00	30	450	0.07
F1	22	0.06	0	0.00	62	0.16	1	0.00	0	0.00	0	0.00	0	0.00	85	393	0.22
F2	19	0.05	0	0.00	62	0.15	0	0.00	0	0.00	0	0.00	0	0.00	81	420	0.19
F3	11	0.03	1	0.00	44	0.11	1	0.00	0	0.00	0	0.00	0	0.00	57	411	0.14
F4	9	0.02	2	0.00	42	0.09	0	0.00	0	0.00	0	0.00	0	0.00	53	477	0.11
F5	12	0.03	6	0.01	70	0.16	2	0.00	10	0.02	0	0.00	2	0.00	102	432	0.24
MIN	2	0.00	0	0.00	21	0.05	0	0.00	0	0.00	0	0.00	0	0.00	30	384	0.07
MAX	31	0.08	40	0.10	70	0.16	11	0.03	10	0.02	0	0.00	2	0.00	131	477	0.34
MEDIAN	11.5	0.03	2	0.00	45	0.12	1	0.00	0	0.00	0	0.00	0.5	0.00	69	436.5	0.17

Distribution of naming errors in the third mapping (second remapping): Summary of naming errors induced by rTMS per subject (M1-M5: male volunteers; F1-F5: female volunteers). Below is reported number of trials (rTMS trains), total number errors, and error type and rate of each given error type of all induced errors.

##### No response errors

148 no response errors were elicited by 4344 stimulations in the left hemisphere. This equals an error rate of 3.4% (Table 5). Again, no response errors were observed in each subject.

##### Performance errors

In contrast to the previous mappings, only 8 out of 10 subjects showed performance errors. Altogether, 61 performance errors were elicited, resulting in an error rate of 1.4% (Table 5).

##### Hesitation errors

Hesitation errors came up to a number of 492. Consequentially, this represents an error rate of 11.3% (Table 5). Hesitations were again observed in all subjects.

##### Other errors

4344 left hemispheric stimulations elicited 24 neologisms (error rate 0.6%) and 13 phonological errors (error rate 0.3%). Furthermore, we obtained 7 semantic errors (error rate 0.2%; Table 5). Again, no circumlocutions were observed.

### Intra- and interobserver reliability

The properties of all three mappings are demonstrated by Table 2. Differences among the mapping parameters did not reach statistical significance.

#### Intraobserver reliability

To determine the intraobserver reliability, the first two mappings, which were conducted by the same examiner, were compared. The reproducibility varied among different error categories. Overall, among intraobserver investigations, no response errors, hesitations, and semantic errors were better reproducible than other error categories. Posterior regions showed lower reproducibility than the anterior regions. Table 6 demonstrates the results expressed by the CCC.

**Table 6:** Concordance correlation coefficient

**Error category**	**All regions**		**Anterior regions**		**Posterior regions**	
	**Intra-observer**	**Inter-observer**	**Intra-observer**	**Inter-observer**	**Intra-observer**	**Inter-observer**
no response	0.356	0.227	0.505	0.147	−0.008	0.038
performance error	−0.047	0.163	0.251	0.049	0.370	−0.008
hesitation error	0.383	0.312	0.288	0.162	0.173	0.161
neologism	−0.035	0.559	0.189	0.238	0.139	0.588
phonological error	---	−0.135	---	−0.292	---	---
semantic error	0.218	0.296	−0.222	0.117	0.038	---

Intra- and interobserver reliability in all areas, in anterior and in posterior regions, expressed by the CCC.

#### Interobserver reliability

To determine the interobserver reliability, the first and third mapping, which were conducted by two different examiners, were compared. Again, we obtained higher reproducibility in anterior regions and in the error categories of “no response”, “hesitations”, and “semantic errors”. Altogether, performance errors and phonological errors showed—as in intraobserver investigations—low reproducibility (Table 6).

## Discussion

During all mapping sessions, and given our previously defined spatial limitations, rTMS was well tolerated by all volunteers. There were neither adverse events nor statistically significant differences concerning the experienced pain. Within our small study group, we conclude that intraprocedural discomfort is not related to the investigator.

The technique of rTMS is a promising new method for preoperative planning of surgery in eloquent cortical areas. In clinical daily routines, it is common practice that a certain examination method is conducted by different investigators. Therefore, high reliability of the respective method is necessary. The authors decided to conduct the remappings blinded to previous mapping setups (frequency, number of pulses, RMT) and results, to both mirror clinical reality and to include the entire rTMS procedure. Our data showed that the rTMS language mapping has limited reliability not only in intra-observer comparisons, but also in interobserver comparisons; although there is not a perfect match, there is a reasonably small range of differences.

The observed variability is ascribable to at least three different causes, though the abovementioned design of the present study does not allow us to determine which of the following three variables has the most important influence on the observed intra- and interobserver variability. First, differences in the performance of the mappings, such as applying distinct mapping parameters, probably lead to different language positive points. Furthermore, reproducibility can also be impaired by the language analysis itself because analyzing the recorded videos is not completely objective. Although our setup with baseline measurements, standardized protocols, and video recording for detailed post-hoc analysis of the language responses reduces a lot of bias, the evaluation of language errors still harbors some subjective issues. Additionally, we have to proceed on the assumption that there exists no absolute stability in the organization of human language function in the brain, which also has a conceivably significant influence on variability in language mapping [27,28].

With regard to the different mapping parameters as a source of mapping differences, the investigators strictly followed the mapping protocol mentioned above during all examinations. According to our mapping protocol, it remains to the investigator to choose between three different setups (5 Hz/5 pulses, 5 Hz/7 pulses, or 7 Hz/7 pulses). Furthermore, the hand knob and the RMT were determined again for each remapping. However, the chosen mapping parameters and the detected RMT show no significant difference concerning both intra- and interobserver investigations (Table 2). Hence, we assume that the choice of parameters is distributed randomly. In other words, none of the examiners seems to prefer a certain mapping setup. Thus, these data suggest that the optimal mapping setup varies considerably even for one subject across the three sessions, yielding a small variability in mapping outcomes.

Concerning the naming error analysis as a source of mapping difference, on one hand, by categorizing the produced errors into pre-defined different error types, the language analysis reaches a certain degree of objectivity. As a result, both the intra- and interobserver variability that may arise at the stage of error analysis could be considered reasonably small. On the other hand, there are still cases in which the investigators are not in accordance. Consequently, it is reasonable that our results demonstrate higher intraobserver compared to interobserver reliability (Figure 2).

When we take a closer look at the intra- and interobserver variability by error types, they were not uniform. Hesitation errors show—besides no response and semantic errors—a high reproducibility. In the existing literature, hesitation errors are suspected to represent a rather untrustworthy error category, which some authors do not even implicate in their analysis [14]. However, our data support the argument that hesitation errors evoked by rTMS should not be ignored; instead, they should be regarded as evidence for disrupted language processing in the brain [1], as is the case with non-navigated rTMS studies that standardly measure naming latency difference to localize specific language functions in healthy adult brains [6].

Another important aspect is the fact that little is known about the stability of language eloquent cortical regions per se. Direct cortical stimulation—the current gold standard for language mapping—assumes that the same stimulated areas do not evoke errors in that region consistently [7]. In the standard DCS language mapping procedure, a cortical site is judged language positive when 2 out of 3 stimulations elicit language errors [9,15]. This 66% criterion means that, even within a very short time lag, absolute reproducibility is impossible even for the gold standard, due to the complex connectivity and therefore plasticity of language function. Furthermore, we have to keep in mind previous studies, which have shown that reorganization of the brain exists not only after strokes or in the course of tumor disease, but also in healthy subjects [29-31].

This natural plasticity may not only be demonstrated for rolandic regions in short-term motor-learning experiments [30,32], cognition, and memory structures in longitudinal real-life extensive subjects learning situation [33], but it also appears in perisylvian eloquent areas and inferior parietal cortex for language perception and memory in a longitudinal code-deciphering learning study. According to the long time lag between first and second or third mapping, plasticity might indeed be a reason for the varying results in the present study. Thus, both intra- and interobserver variability are inevitable.

Additionally, when we take a closer look at the variability by error types, we noticed that, given our protocol, certain error types are better reproducible than others are Table 6). Altogether, in comparison to performance errors, neologisms, and phonological errors, the error categories “no response”, “hesitations”, and “semantic” tend to show higher reproducibility (Table 6). It could be possible that errors that are associated with pronunciation and language production itself exhibit greater fluctuation.

Another explanation could be that, as reported earlier [15], rTMS following the abovementioned mapping protocol seems to be especially useful in anterior sites (Table 6). Compared with posterior sites, anterior language areas tend to demonstrate higher reproducibility and correlation with intraoperative DCS. When reviewing the literature on human language processing, activation of posterior language sites seems to be earlier than 300 ms [1,6]. Therefore, rTMS pulses 300 ms after picture presentation might be too late to disrupt posterior language processing. Thus, when improving the protocol in future investigations, one of the questions we have to ask is how we can evoke more reliable errors in posterior regions, and whether other mapping parameters could provide more reliable results.

Nonetheless, the aim of this study was to gather data about reproducibility of language mapping by rTMS. However, one bias of reproducibility is the spatial inconsistency of the investigated language function itself, which cannot be separated from the inaccuracy of the method. Yet, since is crucial to have data on the reproducibility of a new technique, this study has still its justification. Moreover, being the first study investigating this question, this study intended to mirror current practice of rTMS language mapping rather than examining a new rigid mapping protocol. These further aspects of reproducibility have to undergo further investigation in the future.

## Conclusions

With our current protocol, interobserver and intraobserver comparison only corresponded partially. Although the technique of rTMS seems a promising method for preoperative planning as well as neuropsychological research, the current protocol needs further improvement.

## Abbreviations

CCC: Concordance correlation coefficient; CPS: Cortical parcellation system; DCS: Bipolar direct cortical stimulation; fMRI: functional MRI; MEG: Magnetoencephalography; ISI: Inter-stimulus interval; nTMS: navigated transcranial magnetic stimulation; RMT: Resting Motor Threshold; rTMS: repetitive nTMS; VAS: Visual analogue scale.

## Competing interests

The authors declare that they have no competing interests. The authors declare that they have no conflict of interest affecting this study. The authors report no conflict of interest concerning the materials or methods used in this study, or the findings specified in this paper.

## Authors’ contributions

NS and TH were responsible for data acquisition and drafted the manuscript. AH was responsible for statistical analyses, and read and approved the final manuscript. BM and FR approved and corrected the final version of the manuscript. SK handled the acquired data and performed literature research, as well as statistical analyses. SK drafted the manuscript and its final revision. SK is also responsible for concept and design. All authors read and approved the final manuscript.

## Authors’ information

NS and TH are medical students who are performing a considerable number of rTMS studies. All other authors are strongly involved in the treatment of brain tumors, including awake surgery, preoperative mapping, and intraoperative neuromonitoring in a specialized neurooncological center. BM is chairman, and FR is vice chairman of the department.
